# Asthma diagnosis and treatment – 1012. The efficacy of budesonide in the treatmetn of acute asthma in children: a double-blind, randomized, controlled trial

**DOI:** 10.1186/1939-4551-6-S1-P12

**Published:** 2013-04-23

**Authors:** Abdullah A Alangari, Nidal Malhis, Mohamed Mubasher, Najwa Al-Ghamdi, Mohammed Al-Tannir, Mohammed Riaz, Dale Umetsu, Saleh Al-Tamimi

**Affiliations:** 1 Pediatrics, King Saud University, College of Medicine, Riyadh, Saudi Arabia; 2 Emergency Medicine, King Fahad Medical City, Riyadh, Saudi Arabia; 3 King Fahad Medical City, Saudi Arabia; 4 Pharm D , Pharmacy, King Fahad Medical City, Riyadh, Saudi Arabia; 5 King Fahad Medical City, Riyadh, Saudi Arabia; 6 Harvard University, MA, USA

## Background

Current evidence suggests that inhaled glucocorticoids (IGC) have a more profound topical none genomic effect on bronchial airways as compared to systemic glucocorticoids. The value of adding IGC to current therapy of acute asthma is not well established.

## Methods

We conducted a double-blind, randomized, two-arm, parallel groups, controlled clinical trial to compare the addition of budesonide 1500 mcg or placebo (normal saline) to standard acute asthma treatment (albuterol and ipratropium bromide) administered in 3 divided mixed doses within 1 hour in the emergency department (ED). Children 2-12 years of age with moderate or severe acute asthma, scoring 8-15/15 on a well-validated scoring system were included. Both groups received a single dose of prednisone 2 mg/kg/day (max. 60 mg) at the beginning of therapy. The primary outcome was admission rate within 2-4 hours from starting therapy.

## Results

A total of 723 children were enrolled in the study over 17 months duration, of whom 139 were allowed to re-enroll and be randomized to constitute 906 randomization assignments (458 on the treatment group and 448 on the control group); with baseline mean + SD asthma score of 10.63 + 1.73; age 5.52 + 2.76 years; 35% girls; 30.8% (16.5%) with baseline severe asthma score of ≥12 (≥ 13). Statistical Analysis plan allowed for the potential dependency in response due to reenrollments of a subset of children, using Generalized Linear Mixed Modeling (GLMM) techniques. Baseline demographic and clinical characteristics were not significantly different between the two randomized groups. Seventy-five out of 458 (16.4%) of the treatment group vs. 82/448 (18.3%) of the control group were admitted, (OR 0.85, CI: 0.59-1.23, p-value=0.39). Among the severe asthmatics with baseline score ≥13, treatment vs. placebo group, GLMM adjusted admission rate was 30% vs. 47%, indicating a 17% difference in admission rate in favor of the treatment group (adjusted OR of 0.49, CI: 0.25-0.95; p-value= 0.035) that indicated a 51% reduction in the risk of admission for the treatment vs. control group.

## Conclusions

Children with baseline severe asthma score ≥13 who were treated with budesonide had a significant reduction in their admission rate.

